# Effects of insulin and IGF-I on growth hormone- induced STAT5 activation in 3T3-F442A adipocytes

**DOI:** 10.1186/1476-511X-12-56

**Published:** 2013-04-30

**Authors:** Yuchao Zhang, Yuantao Liu, Xia Li, Weina Gao, Wenjie Zhang, Qingbo Guan, Jing Jiang, Stuart J Frank, Xiangdong Wang

**Affiliations:** 1 The Institute of Cell Biology, Shandong University School of Medicine, Jinan 250012, China; 2 The Second Hospital of Shandong University, Jinan, 250033, China; 3 Shandong Provincial Hospital, affiliated to Shandong University, Jinan, 250021, China; 4 Department of Medicine, Division of Endocrinology, Diabetes, and Metabolism, University of Alabama at Birmingham, Birmingham, AL, 35294, UK; 5 Endocrinology Section, Medical Service, Veterans Affairs Medical Center, Birmingham, AL, 35233, UK; 6 Key Laboratory of Protein Sciences for Chronic Degenerative Diseases in Universities of Shandong (Shandong University), Jinan 250033, China

**Keywords:** GH, Insulin, 3T3-F442A adipocyte, IGF-I

## Abstract

**Background:**

Growth hormone (GH) and insulin signaling pathways are known important regulators of adipose homeostasis. The cross-talk between GH and insulin signaling pathways in mature adipocytes is poorly understood.

**Methods:**

In the present study, the impact of insulin on GH-mediated signaling in differentiated 3T3-F442A adipocytes and primary mice adipocytes was examined.

**Results:**

Insulin alone did not induce STAT5 tyrosine phosphorylation, but enhanced GH-induced STAT5 activation. This effect was more pronounced when insulin was added 20 min prior to GH treatment. The above results were further confirmed by *in vivo* study, showing that insulin pretreatment potentiated GH- induced STAT5 tyrosine phosphorylation in visceral adipose tissues of C57/BL6 mice. In addition, our *in vitro* results showed that IGF-I had similar potentiating effect as insulin on GH-induced STAT5 activation. *In vitro*, insulin and IGF-I had an additive effect on GH- induced MAPK activation.

**Conclusion:**

These results indicate that both insulin and IGF-I specifically potentiated GH mediated STAT5 activation in mature adipose cells. These findings suggest that insulin and GH, usually with antagonistic functions, might act synergistically to regulate some specific functions in mature adipocytes.

## Background

Growth hormone (GH) is a 22-kDa peptide that plays important roles in regulation of growth and metabolism [1,2]. Binding of GH to its receptor (GHR), a transmembrane glycoprotein member of the cytokine receptor superfamily [2], results in receptor dimerization and rapid activation of the tyrosine kinase Janus kinase 2 (JAK2) [3]. This in turn initiates a variety of signaling cascades, including the signal phosphatidylinositol 3-kinase (PI3K) and mitogen-activated protein kinases (MAPK/ERK) pathways [4]. In addition to PI3K and MAPK, GH has been shown to activate the STATs mediated signaling pathways [2]. In particular, STAT5a and b are believed to be the major downstream targets of GH [5]. STAT5a and b are encoded by different genes, however, they share > 90% amino acid identity [6]. Upon GH stimulation, STAT5 were recruited to GHR and subsequently phosphorylated on tyrosine residues [6]. Tyrosine phosphorylated STAT5s dimerize and translocate to the nucleus where it binds to specific DNA elements to modulate gene expression in fat tissue in response to GH [6-10]. Studies on the identification of STAT5 target genes in adipocytes indicate that STAT5 modulate gene expression in a manner that favors a reduction in lipid synthesis and/or storage and an increase in lipid release [11-13]. In rat pre-adipocytes, the ability of GH to inhibit expression of aP2, a fatty acid-binding protein in adipocytes, is largely dependent on the GH-induced activation of STAT5 [14]. Growth hormone stimulated lipolysis has been confirmed by evidence from STAT5a and b knockout mice [15].

Insulin is the key regulatory hormone in control of glucose homeostasis and metabolism in muscle and adipose tissue [16]. Insulin acts via binding to its cell surface receptor, and subsequently induces the phosphorylation of insulin receptor substrate proteins (IRSs) [17]. Downstream of IRSs, the major signal pathways activated by insulin are PI3K and MAPK pathways [17,18]. In adipose tissue, insulin was shown to increase the adipocyte size through activation of PI3K [18].

The above studies indicate that both GH and insulin play important roles in regulation of adipocyte functions. Moreover, GH and insulin signaling pathways share some downstream elements. However, the interactions and signal integration between these two signaling pathways in mature adipocytes is poorly understood. In the present study, we determined the effects of insulin on GH mediated signaling pathways in adipocytes both *in vitro* and *in vivo*.

## Results

### Effects of GH, insulin and IGF-I on STAT5 and MAPK activation in differentiated 3T3-F442A adipocytes

**Figure 1:**
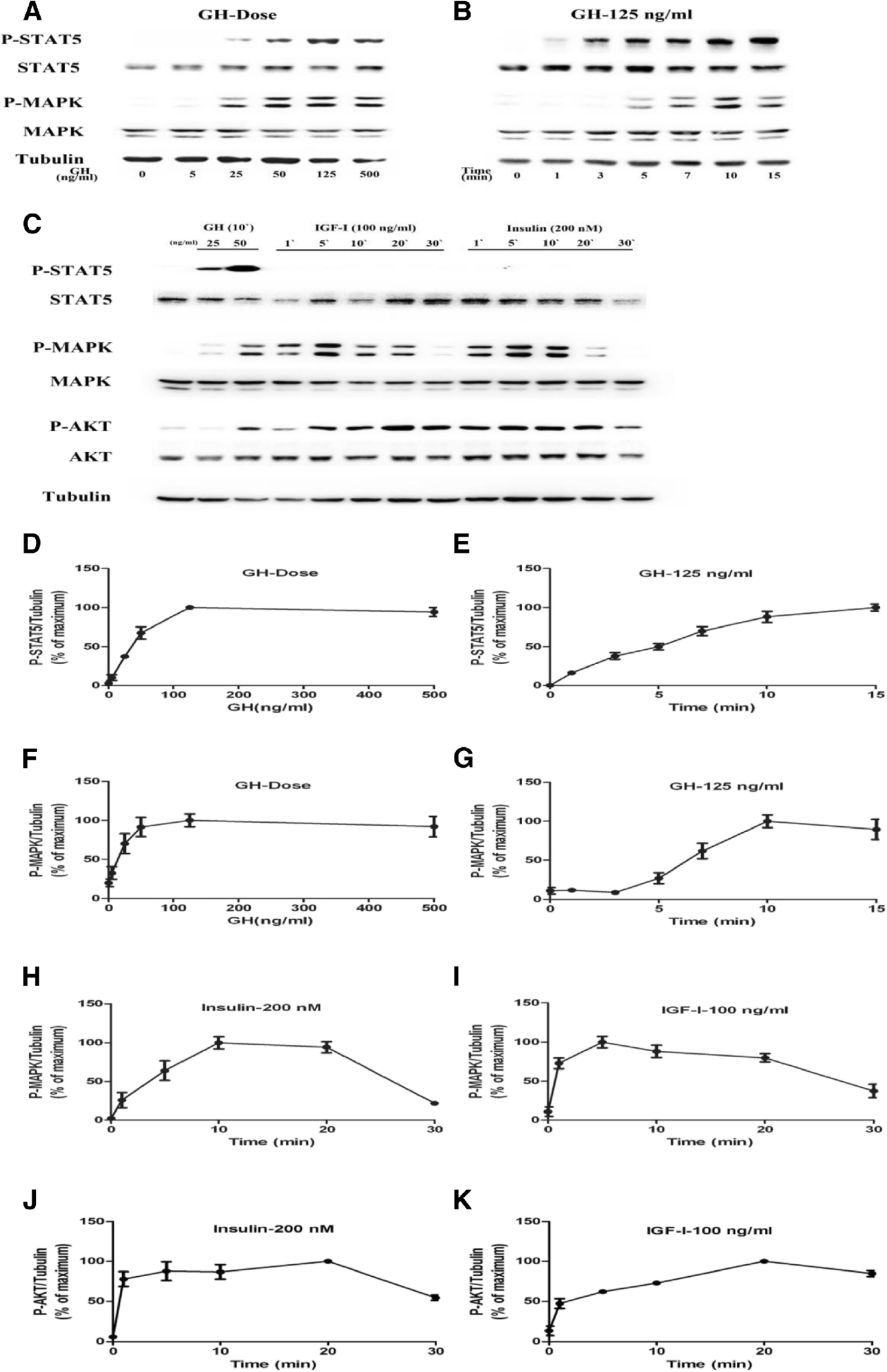
**GH and insulin- induced STAT5 and MAPK activation in differentiated 3T3-F442A adipocytes. **STAT5, MAPK and AKT activations were analyzed by Western blot using specific antibodies against phosphorylated forms of STAT5, MAPK and AKT (**A**-**C**). Densitometric analysis of the results from Western Blots (**D**-**K**). **A**, **D** and **F**, STAT5 and MAPK activation induced by GH at various concentrations for 10 min (n = 4). **B**, **E **and **G**, STAT5 and MAPK activation induced by a single dose of GH (125 ng/ml) for indicated times (n = 4). **C**, **H **to **K**, STAT5 MAPK and AKT activation induced by IGF-I (100 ng/ml) or insulin (200 nM) (n = 3).

3T3-F442A pre-adipocytes were induced to differentiate as previously described [19], and cell differentiation was confirmed by Oil-Red-O staining. Differentiated 3T3-F442A cells were stimulated with GH, insulin or IGF-1 respectively. Results of western blot showed that GH induced STAT5 tyrosine phosphorylation in differentiated 3T3-F442A cells in dose- and time- dependent manners (Figure 1A,B). A maximum STAT5 phosphorylation was induced by 125 ng/mL GH for 15 min (Figure 1D,E). Similarly, GH also induced MAPK activation in dose- and time- dependent manners (Figure 1A,B,F,G). A maximum MAPK activation was induced by 125 ng/mL GH for 10 min.

Under same conditions, insulin (200 nM) or IGF-I (100 ng/mL) induced maximum MAPK activation at 10 min (Figure 1C,H) or 5 min (Figure 1C,I) respectively. Similarly, Insulin and IGF-I also induced the phosphorylation of AKT (Figure 1C,J,K). However, Insulin and IGF-I did not induce detectable STAT5 tyrosine phosphorylation.

### Effects of insulin on GH-induced STAT5 and MAPK activation in differentiated 3T3-F442A adipocytes

**Figure 2:**
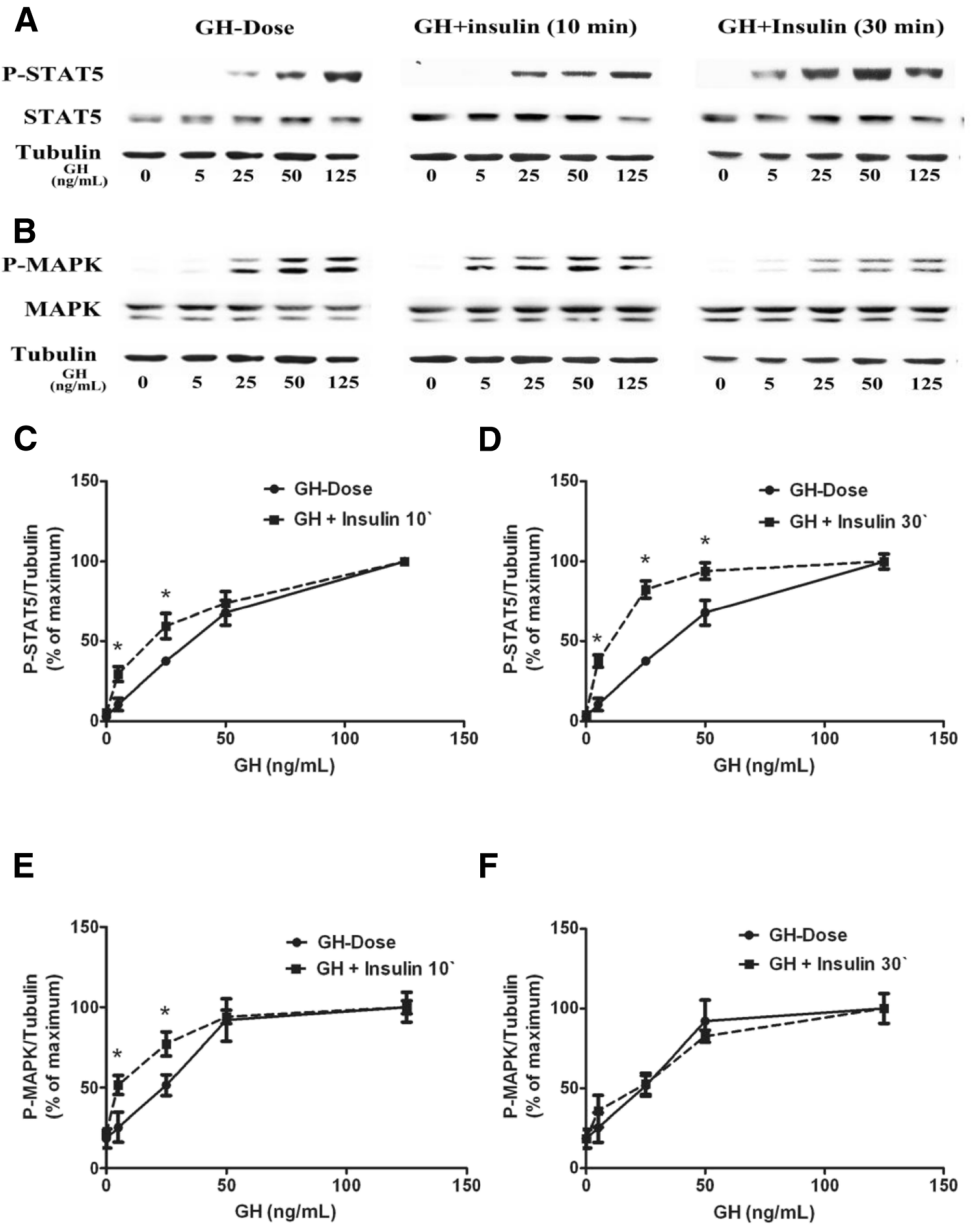
**Effects of insulin on GH- induced STAT5 and MAPK activation in differentiated 3T3-F442A adipocyte cells. A**, **B**, representative results of Western blot (n = 4). **C**, GH versus GH plus insulin (10 min) on STAT5 activaion (n = 6). **D**, GH versus insulin pretreatment (insulin was added 20 min prior to GH) on STAT5 activation (n = 6). **E**, **F**, GH versus GH plus insulin on MAPK activation. ^*^p < 0.05, *vs. *GH alone.

**Figure 3:**
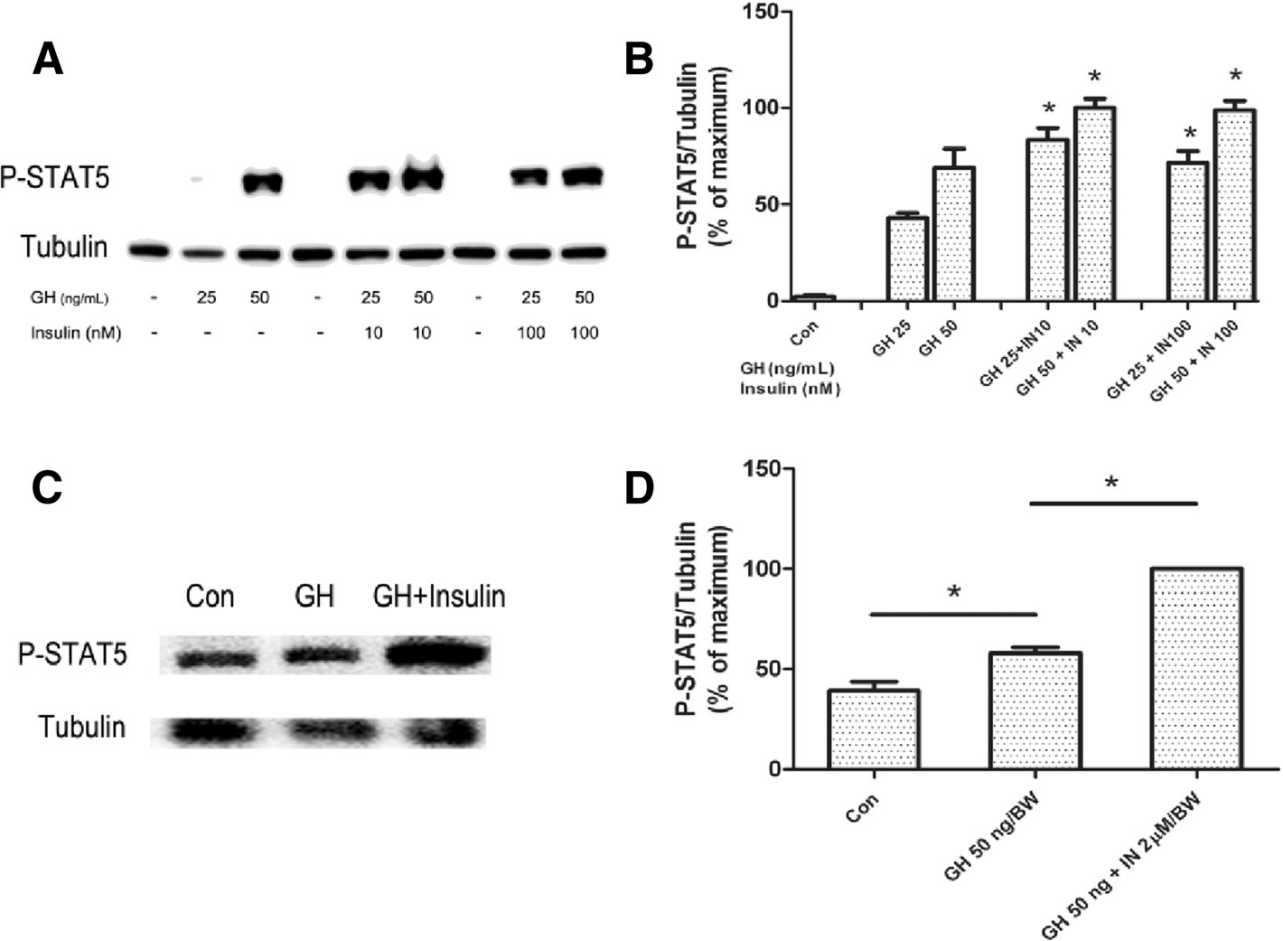
**Insulin pretreatment on GH induced STAT5 activation *in vitro *(A, B) and *in vivo *(C,D). A**, **B**, pretreatment with 10 nM or 100 nM insulin (insulin was added 20 min prior to GH) on GH- induced STAT5 activation in differentiated 3T3-F442A cells (n = 4). **C**, **D**, visceral adipose tissues around the kidneys were extracted from C57/BL6 mice with GH (50 μg/kg. BW), GH (50 μg/kg. BW) + insulin (2 μM/kg. BW injected prior 20 min to GH) or saline injection (n = 5). ^*^p < 0.05, GH alone *vs.*control; GH + insulin *vs. *GH alone.

Differentiated 3T3-F442A cells were treated with GH (0, 5, 25, 50 and 125 ng/mL) or co-treated with insulin (200 nM) for 10 min, or pretreated with 200 nM insulin for 20 min followed by addition of GH for additional 10 min. Results showed that simultaneous insulin treatment significantly increased STAT5 phosphorylation compared to GH alone (Figure 2A,C). Insulin pretreatment for 20 min further increased GH-induced STAT5 phosphorylation compared to GH and insulin co-treatment (Figure 2A,D). As insulin at high concentration may activate IGF-I receptor, thus we examined the effects of insulin at lower doses (close to physiological concentrations) on GH-induced STAT5 activation. As shown in Figure 3A/B, insulin at lower doses (10 nM and 100 nM) also enhanced GH-induced STAT5 phosphorylation (Figure 3A,B).

Under same conditions, the effects of insulin on GH-induced MAPK activation were examined. Simultaneous insulin (200 nM) treatment had an additive effect on GH- (5 ng/mL, 25 ng/mL) induced MAPK activation (Figures 2B,E). Insulin (200 nM) pretreatment (insulin was added 20 min prior to GH) did not influence GH-induced MAPK activation (Figure 2B,F).

### Insulin enhanced GH-induced STAT5 activation in primary adipocytes from C57/BL6 mice

C57/BL6 mice were pretreated with or without insulin (2 μmol/kg.BW) injection for 20 min, and then were injected with GH (50 μg/kg.BW). 10 min after GH injection, mice were sacrificed, and visceral adipose tissues were isolated for western blot analysis. Insulin pretreatment increased STAT5 phosphorylation in adipose tissues by 60% as compared to mice treated with GH alone (Figure 3C,D). GH induced MAPK activation was not influenced by insulin pretreatment (data not shown).

### Effects of IGF-I on GH-induced STAT5 and MAPK activation in differentiated 3T3-F442A adipocytes

**Figure 4:**
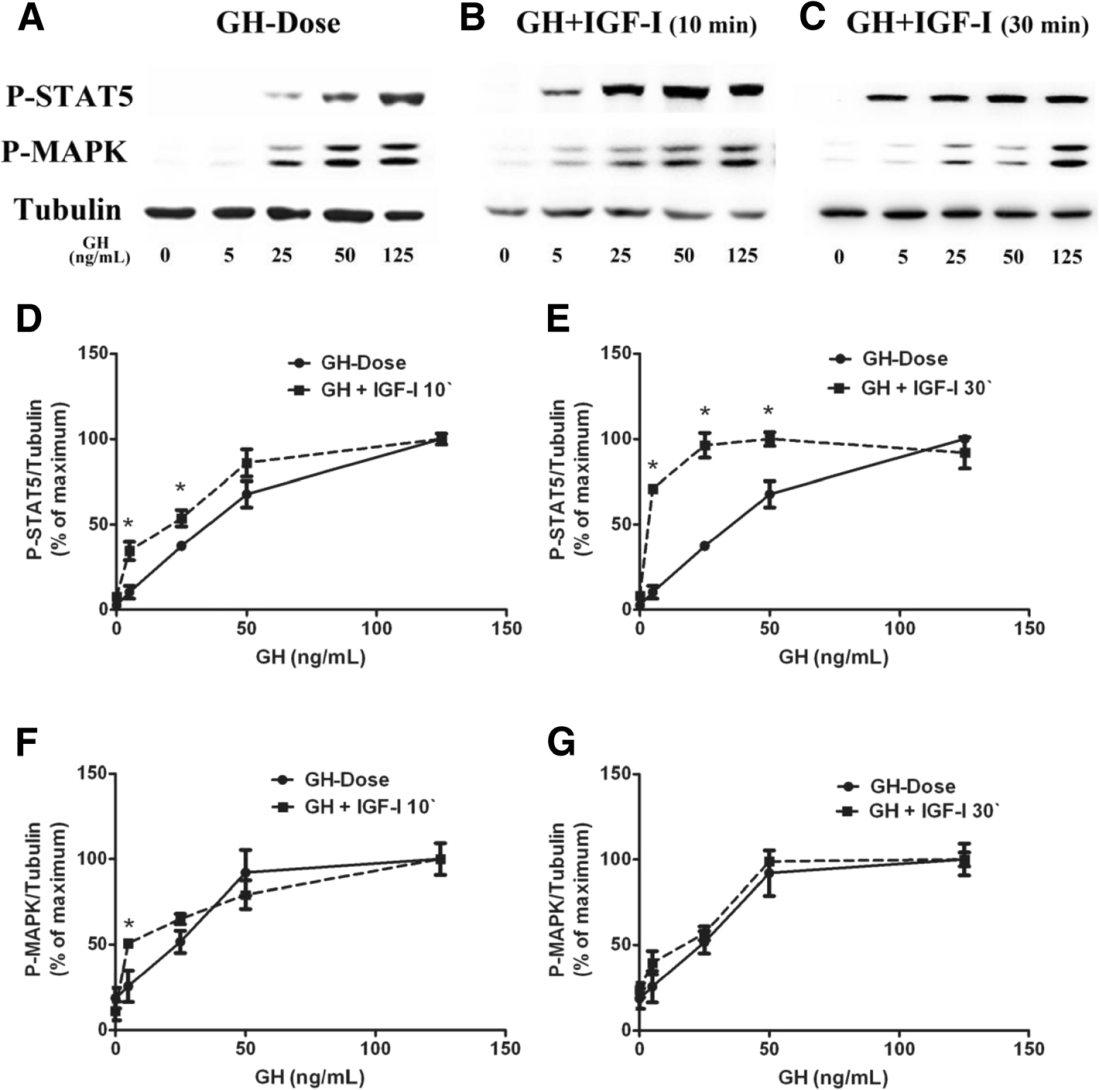
**Effects of IGF-I on GH -induced STAT5 and MAPK phosphorylation in differentiated 3T3-F442A adipocytes. A**, **B**, **C**, representative results of Western blot (n = 4). **D**, GH versus GH plus IGF-I (10 min) on STAT5 activation (n = 4). **E**, GH versus IGF-I preatreatment (IGF-I was added 20 min prior to GH) on STAT5 activation (n = 4). **F**, GH versus GH plus IGF-1 (10 min) on MAPK activation (n = 4). **G**, GH versus IGF-I pretreatment (IGF-I was added 20 min prior to GH) on MAPK activation (n = 4). ^*^p < 0.05, *vs.* GH alone.

As IGF and insulin have similarities in their mode of signaling and functions, thus we tested whether IGF-I has similar effects as insulin on GH mediated signaling in mature adipocytes. Differentiated 3T3-F442A cells were treated with GH alone or co-treated with IGF-I (100 ng/mL) for 10 min; or pretreated with IGF-I (100 ng/mL) for 20 min followed by GH stimulation for additional 10 min. Simultaneous IGF-I treatment enhanced GH (5 ng/mL) induced STAT5 and had an additive effect on GH induced MAPK activation (Figure 4A,B,D,F). IGF-I pretreatment for 20 min enhanced STAT5 activation induced by GH of varying concentrations (GH 5, 25, 50 ng/mL) (Figure 4C,E); but did not change the profile of MAPK (Figure 4A,C,G).

## Discussion

Both growth hormone and insulin play essential roles in control of development and metabolism. At the whole body level, the actions of these two hormones are highly coordinated. For instance, in the early postprandial phase, increase in insulin secretion and decrease in GH secretion favors the disposal of glucose and fat; whereas in the post-absorptive phase, increase in GH secretion and decrease in insulin promote lipolysis and fat oxidation [20]. At the cellular level, however, the functions of GH and insulin are distinct and usually antagonistic. In adipocytes, the direct interactions between these two hormones are not well understood.

Previous studies indicated that GH could induce lipolysis in adipose tissue [10,12], which is associated with STAT5 activation. In the present study, we demonstrated that GH induced STAT5 activation in dose- and time- dependent manners in mature 3T3-F442A adipocytes. In addition, our results showed that insulin alone had no detectable effect on STAT5 tyrosine phosphorylation, but significantly enhanced GH-induced STAT5 tyrosine phosphorylation. Our results suggested that insulin had a potentiating effect on GH induced STAT5 activation.

To determine whether the potentiating effect of insulin is specific for STAT5, we next examined the effect of insulin on GH induced MAPK activation. Our results showed insulin had an additive effect rather than potentiating effect on GH-induced MAPK activation.

To confirm our *in vitro* results, we conducted animal experiments using C57/BL6 mice. As female mice have relatively more stable plasma GH levels [21], therefore female C57/BL6 mice were used in the present study. The results showed that insulin enhanced GH-induced STAT5 tyrosine phosphorylation in primary mice adipocytes, but had no obvious effect on GH induced MAPK activation (result did not show).

As IGF-I and insulin have similarities in their mode of signaling and functions, thus we tested whether IGF-I has similar effects as insulin on GH mediated signaling in mature adipocytes. Similar to insulin, IGF-I showed a potentiating effect on GH- induced STAT5 activation but had an additive effect on GH- induced MAPK activation. These findings suggested insulin and IGF-I might produce some common signals, which specifically potentiate GH-induced STAT5 tyrosine phosphorylation. Interestingly, a previous study has shown that chronic GH treatment of differentiated 3T3-L1 adipocytes reduces insulin-stimulated 2-deoxyglucose (DOG) uptake and activation of AKT, leading to insulin resistance [22]. So far, the acute effect and significance of GH on insulin signaling have not been well documented.

To date, the mechanism behind the differential effects of insulin on GH induced STAT5 and MAPK activation is unclear. There is evidence showing that GHR, insulin receptor, IGF-I receptor were co-localized in lipid rafts [23-26]. It is possible that lipid raft may provide a platform for the interactions between these signaling molecules.

## Conclusions

In summary, our results showed that insulin specifically potentiates GH-induced STAT5 activation in mature adipocytes both *in vitro* and *in vivo*. These findings suggested that insulin and GH, usually with antagonistic functions, might act synergistically to regulate some specific functions in mature adipocytes. The downstream biological events and clinical relevance of the interactions between insulin and GH need further investigation.

## Methods

### Materials

Recombinant human GH was kindly provided by Eli Lilly & Co. (Indianapolis, IN). Recombinant human insulin was from Sigma (St. Louis, MO, USA). IGF-I was purchased from NovozymesGroPep Ltd (Thebarton, South Australia, Australia). Cell culture medium and fetal bovine serum (FBS) were from Hyclone (ThermoScientific, Inc., Illinois, USA). All other reagents were purchased from Sigma.

### Antibodies

Polyclonal antibody against phospho-tyrosine-STAT5 was purchased from Invitrogen (CA, USA); polyclonal antibody against STAT5 was purchased from Santa Cruz Biotechnology, Inc (Santa Cruz, CA); polyclonal antibody against phospho- ERK (recognizing the dually phosphorylated Thr-183 and Tyr-185 residues corresponding to the active forms of ERK1 and ERK2) was from Promega (Madison, WI); affinity-purified polyclonal antibody against ERK (recognizing both ERK1 and ERK2) was from Upstate Biotechnology (Lake Placid, NY); polyclonal antibody against AKT and phospho-AKT (S473) were purchased from Cell Signaling Technology (Beverly, MA, USA) and monoclonal antibody against α-tubulin was from Sigma. All secondary antibodies were purchased from ZhongShanJinQiao (China).

### Cell culture

3T3-F442A cells, kindly provided by Drs. H. Green (Harvard University, Boston, MA) and C. Carter-Su (University of Michigan, Ann Arbor, MI), were cultured and induced to differentiate as described previously [19]. In brief, cells were cultured in Dulbecco’s modified Eagle’s medium containing (DMEM) 4.5 g/L glucose, supplemented with 10% new born calf serum (NCS), 50 μg/mL streptomycin and 100 U/mL penicillin until they reached confluence. To induce differentiation, confluent cells were cultured in DMEM containing 10% FBS with 5 μM insulin. The differentiation medium was refreshed every other day. After 3 times of medium changes, insulin was removed from the culture medium. Cells were cultured with DMEM containing 10% FBS, and the medium was refreshed every other day. After 12 days, approximately 80% cells were differentiated into adipocytes as confirmed by Oil Red- O staining (not shown).

### Cell stimulation

Differentiated F442A adipocytes were starved in culture medium containing 0.5% (w/v) bovine serum albumin (BSA) for 16 h before stimulation. All the stimulations were carried out at 37°C in DMEM with 0.5% BSA. For dose–response experiments, serum-starved cells were treated with: GH (0, 5, 25, 50, 125, or 500 ng/mL) for 10 min; GH (0, 5, 25, 50, or 125 ng/mL) plus 200 nM insulin for 10 min; GH in the presence of insulin (10, 100 or 200 nM) for 30 min (added 20 min prior to GH); GH plus 100 ng/mL IGF-I for 10 min; or GH plus IGF-I for 30 min (100 ng/mL IGF-I was added 20 min prior to GH). For time course experiments, serum-starved cells were treated with: GH (125 ng/mL) for different periods (0, 1, 3, 5, 7, 10 or 15 min), IGF-I (100 ng/mL) or insulin (200 nM) for different periods (0, 1, 5, 10, 20 or 30 min). Stimulation was end by washing cells twice with an ice-cold phosphate-buffered saline (PBS) in presence of 0.4 mM sodium orthovanadate.

### Animal experiments

Female C57/BL6 mice, aged 12-weeks, weighing 20–24 g, were purchased from the Experimental Animal Center of Shandong University. Animal experiments were carried out according to the ‘Principles of laboratory animal care’ established by the National Institutes of Health, and approved by the ‘Animal Care and Use Committee’ of the Shandong University (Number: MECSDUMS 2011055). Mice were maintained under diurnal lightning conditions at 25°C with free access to tap water and food. Mice were randomly divided into three groups: the “control group”, “GH group”, and “GH + insulin group”. GH group mice were injected intraperitoneally (i.p.) with 50 μg of GH per kg of body weight (BW) in 0.2 ml saline for 10 min before sacrifice. GH + insulin group mice were first injected i.p. with 2 μmol insulin/kg.BW in 0.1 mL saline and 20 min later with 50 μg/kg.BW GH in 0.1 mL saline before sacrifice 10 min thereafter. The control mice were injected with saline. Prior to injection, all mice were fasted overnight. After treatment, mice were sacrificed by decapitation under anesthesia with 10% chloral hydrate. Visceral adipose tissues around the kidneys were collected for western blot analysis.

### Western blotting

Cells or adipose tissues were harvested and solubilized for 60 min on ice in lysis buffer (150 mM NaCl, 10% (vol/vol) glycerol, 50 mM Tris-HCl (pH 7.3), 1 mM EDTA, 1.5 nM magnesium chloride, 10 mM sodium pyrophosphate, 2 mMphenylmethylsulphonyl fluoride (PMSF), 100 mM sodium fluoride, 1 mM sodium orthovanadate, with 1% (wt/vol) Triton X-100. Protein concentration was determined by Bradford method (BCA protein assay reagent, Beyotime, China). Equal amounts of protein were separated by SDS-polyacrylamide gel electrophoresis and electro-transferred onto a polyvinylidenedifluoride (PVDF) membrane. Proteins were detected by immunoblotting imaged with ECL chemiluminescence (Amersham Biosciences, Buckinghamshire, England).

### Densitometric analysis

Chemiluminescence signals were detected with a lumino-image analyzer Fluor Chem E (Alpha View, Santa Clara, CA 95051). Densitometric quantification of images exposed in the linear range was performed using an image analysis program, Image J (developed by W. S. Rasband, Research Services Branch, National Institute of Mental Health, Bethesda, MD).

### Statistical analysis

All data are expressed as mean ± SEM. Differences between groups were examined by Student’s *t* test or ANOVA using SPSS17.0 software. A value of *p*< 0.05 was considered statistically significant.

## Competing interests

The authors declare that they have no competing interests.

## Authors’ contributions

YCZ and YTL conducted research and wrote paper. WNG and QBG performed the statistical analysis. XL, JJ and WJZ helped in conducting research. XDW and SJF designed the research. All authors read and approved the final manuscript.
